# Molecular Characterization, Nutritional and Insulin Regulation of Elovl6 in Rainbow Trout (*Oncorhynchus mykiss*)

**DOI:** 10.3390/biom10020264

**Published:** 2020-02-10

**Authors:** Yongnan Li, Yuning Pang, Zengqi Zhao, Xiaojun Xiang, Kangsen Mai, Qinghui Ai

**Affiliations:** 1 Key Laboratory of Aquaculture Nutrition and Feed (Ministry of Agriculture) & Key Laboratory of Mariculture (Ministry of Education), Ocean University of China, 5 Yushan Road, Qingdao 266003, China; nanyongli2013@163.com (Y.L.); 15530793072@163.com (Y.P.); Zhaozengqi123456@163.com (Z.Z.); xxj_ouc@163.com (X.X.); kmai@ouc.edu.cn (K.M.); 2 Laboratory for Marine Fisheries Science and Food Production Processes, Qingdao National Laboratory for Marine Science and Technology, 1 Wenhai Road, Qingdao 266237, China

**Keywords:** rainbow trout, *elovl6*, fatty acids, insulin

## Abstract

Elongation of very long-chain fatty acids protein 6 (Elovl6) is a crucial enzyme in the synthesis of endogenous fatty acids, which participates in the energy balance and metabolic diseases. The main objective of this study was to explore the molecular characterization of Elovl6 and the regulation of *elovl6* expression in response to dietary fatty acids and insulin. In the present study, the ORF (open reading frame) of Elovl6 from rainbow trout was cloned and characterized, which showed a high identity (87%) with mammals and other teleost. The results of quantitative PCR showed that the transcriptional levels of *elovl6* from rainbow trout that were fed diets containing soybean oil (enriched with 18:2n-6, linoleic acid (LA)) or linseed oil (enriched with 18:3n-3, α-linolenic acid (ALA)) were lower than those in the group that were fed diets containing fish oil (enriched with 20:5n-3, eicosapentaenoic acid (EPA) and 22:6n-3, docosahexaenoic acid (DHA)). Correspondingly, mRNA expression of *elovl6* in hepatocytes treated with DHA was dramatically higher than that in LA and ALA groups. The transcriptional expression of *elovl6* in hepatocytes treated with insulin was also significantly increased. Moreover, the dual luciferase assay showed the transcription factor CREB1 dramatically up-regulated the promoter activity of *elovl6*, while FOXO1 significantly down-regulated the *elovl6* promoter activity in rainbow trout. The differences in transcriptional expression of *crbe1* and *foxo1* may contribute to the increase or decrease of *elovl6* expression in rainbow trout in response to fatty acids or insulin. These findings revealed the molecular characterization of *elovl6* and the regulation of *elovl6* expression by CREB1 and FOXO1 in rainbow trout in response to dietary fatty acids or insulin.

## 1. Introduction

De novo lipogenesis (DNL) is a process of converting excess carbohydrates into fatty acids and triglycerides, which is an essential and complex physiological process [1]. As the unique and last elongase involved in the synthesis of endogenous fatty acids in DNL, elongation of very long-chain fatty acids protein 6 (Elovl6) acted to catalyze the elongation of palmitate (PA, C16:0) or palmitoleic acid (POA, C16:1n-7) to stearate (C18:0) or vaccenic acid (C18:1n-7) [2,3]. Recent studies have suggested that Elovl6 is related to energy balance and metabolic diseases, which could be regulated by nutrients and hormones [4].

Elovl6 was highly expressed in the liver, brain, and adipose tissue, which played a crucial role in maintaining the metabolic balance of the fatty acids [5]. Loss of Elovl6 function reduced the concentrations of C18:0 and C18:1n-7, but increased C16:0 and C16:1n-7 contents in mice (*Mus musculus*) [6], and dietary fatty acids (PUFAs) could profoundly regulate the expression of *elovl6* in mice [5]. In the large yellow croaker (*Larimichthys crocea*), *elovl6* expression was increased with the replacement of fish oil by soybean oil or linseed oil in diets via the regulation of transcription factors including hepatocyte nuclear factor 1α (HNF1α), CCAAT-enhancer-binding protein β (CEBPβ), retinoid X receptor α (RXRα), and cAMP response element-binding protein (CREB1) [7].

Furthermore, Elovl6 also showed a crucial function for insulin resistance [3]. The deletion of *elovl6* in mice could prevent diet-induced insulin resistance, and specific deletion of *elovl6* in leptin receptor-deficient mice increased insulin adaptability [3,8]. However, in aquatic animals, Elovl6 was only cloned in *Misgurnus anguillicaudatus* [9], *Eriocheir sinensis* [10], and *Larimichthys crocea* [7] to date, the regulation of dietary fatty acids or insulin on *elovl6* expression is still poorly understood.

Rainbow trout (*Oncorhynchus mykiss*) is an essential commercial freshwater fish that has been widely farmed around the world. The objective of the present study was to investigate the molecular characterization of *elovl6*, and the regulatory mechanism of *elovl6* expression in response to dietary fatty acids or insulin in rainbow trout. The results may strengthen the understanding of nutritional and hormonal regulation, and add new data about the regulatory mechanism of the metabolism in rainbow trout, which could aid in the healthy and sustainable wide aquaculture of rainbow trout.

## 2. Materials and Methods

### 2.1. Animal Experiments

The present study was carried out in strict accordance with the Management Rule of Laboratory Animals (Chinese Order No. 676 of the State Council, revised 1 March 2017). With fish oil, soybean oil, and linseed oil as the lipid source, three isoproteic and isolipidic diets (43% crude protein and 12% crude fat) were prepared and labeled as fish oil (FO), soybean oil (SO), and linseed oil (LO). Fish fasted for 24 h before the experiment. The fish with similar size (mean weight 10.03 ± 0.02 g) were obtained from a commercial farm in Weifang, Shandong, China, and randomly assigned to nine tanks in groups of 30 fish each with continuous flow-through freshwater supply and aeration, with water temperature maintained at 18 ± 2 °C and dissolved oxygen at 7–8 mg/L. Triplicate groups of fish were randomly allocated to FO, SO, and LO, respectively. The fish were fed with each diet to satiation by hand twice daily at 06:00 and 18:00, respectively, for ten weeks. At the end of the experiment, liver, muscle, intestine, heart, spleen, gill, eye, brain, and adipose tissue were collected for RNA isolation and gene expression analyses after fish fasted for 24 h.

### 2.2. RNA Isolation and cDNA Synthesis

Total RNA extraction and cDNA synthesis were performed by TransZol (TransGen Biotech, Beijing, China) and cDNA by the PrimeScriptTM RT reagent kit (Takara, Japan) according to the manufacturer’s instructions.

### 2.3. Cloning, Sequence, and Phylogenetic Analysis of Elovl6

The cloning primers of *elovl6* were designed based on the sequence of rainbow trout on NCBI (XM_021584104.1) (Table 1). Primers were synthesized by Sangon Biotech Co., Ltd., Shanghai, China. PCR was performed in PrimeSTAR^®^Max DNA Polymerase (Takara, Japan) and the total system was 25 μL (1 μL liver cDNA, 1 μL each primer, 12.5 μL PrimeSTAR^®^Max, and 9.5 μL DEPC water). PCR amplification was carried out for 35 cycles under the following procedure: 98 °C for 10 s, 55 °C for 15 s, and 72 °C for 1 min, followed by 72 °C for 10 min. The PCR products were separated by electrophoresis on a 1.0% agarose gel, purified and ligated into the pEASY-T1 vector (TransGen, Beijing, China) for further sequencing (Sangon Biotech Co., Ltd., Shanghai, China). Both the nucleotide sequence and the amino acid sequence of Elovl6 were analyzed on the NCBI blast program (https://blast.ncbi.nlm.nih.gov/Blast.cgi). The multiple sequence alignment was performed by DNAMAN (Lynnon BioSoft, Vaudreuil, Quebec, Canada) and ESPript 3.0 (http://espript.ibcp.fr/ESPript/cgi-bin/ESPript.cgi). The phylogenetic analyses were constructed by the MEGA6 program and the tree was obtained by the neighbor-joining method.

### 2.4. Real-Time Quantitative PCR (RT-qPCR) Analysis

qPCR of the target genes including *elovl6*, forkhead transcription factor (*foxo1*), and *creb1* in rainbow trout was performed in a quantitative thermal cycler (CFX96TM Real-Time System, BIO-RAD, Hercules, CA, USA) by a SYBR Green real-time PCR kit (Takara, Japan). The primers for RT-qPCR were designed by Primer Premier 5.0 (Premier Biosoft, San Francisco, CA, USA) based on the cloned nucleotide sequences, which are listed in Table 1. The results of real-time quantitative experiments were normalized to that of β-actin by the 2^−ΔΔCt^ method [11].

### 2.5. Cloning of Elovl6 Promoter from Rainbow Trout

The genomic DNA of rainbow trout was isolated by the FastPure Cell/Tissue DNA Isolation Mini Kit (Vazyme, Nanjing, China) according to the manufacturer’s instructions. The promoter sequence of *elovl6* was cloned by genomic DNA and inserted into the pGL3-basic luciferase reporter plasmid (Promega, Madison, WI, USA). The plasmid was extracted by the Easypure^®^Hipure Plasmid MiniPrep Kit (TransGen, Beijing, China). JASPAR (http://jaspar.genereg.net/) and TF binding (http://tfbind.hgc.jp/) were used to predict potential transcription factor binding sites in the promoter of *elovl6* in rainbow trout. We set up the relative profile score threshold as 80% and selected the transcription factors with high scores and conservative binding sites to study in the present study.

### 2.6. Construction of Transcription Factor Plasmids, Cell Culturing, Transfection, and Luciferase Assay

For transcription factor plasmids, the ORFs (open reading frames) of sterol regulatory element-binding protein 1 (SREBP1, XM_021624594.1), sterol regulatory element-binding protein 2 (SREBP2, XM_021625095.1), HNF1α (XM_021621309.1), CCAAT-enhancer-binding protein α (CEBPα, NM_001172496.1), CEBPβ (NM_001124447.1), CREB1 (XM_021597386.1), FOXO1 (XM_021618954.1), RXRα (XM_021590263.1), P65 (XM_021578194.1), and peroxisome proliferator-activated receptor γ (PPARγ, XM_021610844.1) were cloned into PCS2^+^ plasmids (Invitrogen, Carlsbad, CA, USA) by the ClonExpress II One Step Cloning Kit (Vazyme, Nanjing, China).

HEK 293T cells were cultured according to the method described in the previous studies [12]. Transfection was performed using Lipofectamine 3000 (Invitrogen, Carlsbad, CA, USA) according to the instructions. A total of 200 ng of promoter reporter plasmid, 600 ng of transcription factor plasmid, 20 ng of pRL-TK renilla luciferase, and 2.0 μL Lipofectamine 3000 were co-transfected in the 24-well plate in triplicate wells with three independent experiments. The TransDetect Double-Luciferase Reporter Assay Kit (TransGen, Beijing, China) and InfiniTE200 plate reader (Tecan, Männedorf, Switzerland) were used for the detection of dual luciferase activity.

### 2.7. Primary Hepatocyte Isolation and Incubation

The primary hepatocyte of rainbow trout was isolated by the perfusion method. Rainbow trout fasted for 24 h before the experiment. After anesthesia with MS-222, the fish was dissected to expose the liver tissue. Hanks (Solarbio, Beijing, China) was injected into the hepatic portal vein until the liver became utterly white. Then, collagenase was pushed slowly into the liver for 30 min. The liver was cut into small fragments, centrifuged at 700× *g* for 5 min after filtration, and the obtained cells were washed with complete medium (DMEM-F12 + 15% FBS). Then the cells were diluted to a certain concentration and stored at 18 °C. Fatty acids, including linoleic acid (LA), α-linolenic acid (ALA), eicosapentaenoic acid (EPA), and docosahexaenoic acid (DHA) (Cayman Chemical Co., Ann Arbor, MI, USA), were supplemented in the form of BSA/fatty acid complexes at 100 μM for 12 h in triplicate wells with three independent experiments. Furthermore, the primary hepatocyte of rainbow trout was treated with insulin (1 nM, 10 nM, and 100 nM, Solarbio, Beijing, China) for 6 h and 8 h in triplicate wells with three independent experiments. The concentrations and incubation times of fatty acids and insulin were determined by a preliminary experiment. After incubation, cells were lysed in the wells and harvested for RNA extraction.

### 2.8. Statistical Analysis

Statistical analysis of all data was performed using SPSS 20.0 and the results were presented as means ± S.E.M. One-way ANOVA and Tukey’s test were used to inspect the differences among more than two groups, and the differences between two groups were determined by Student’s *t* test.

## 3. Results

### 3.1. Molecular Characterization of Rainbow Trout Elovl6

**Figure 1:**
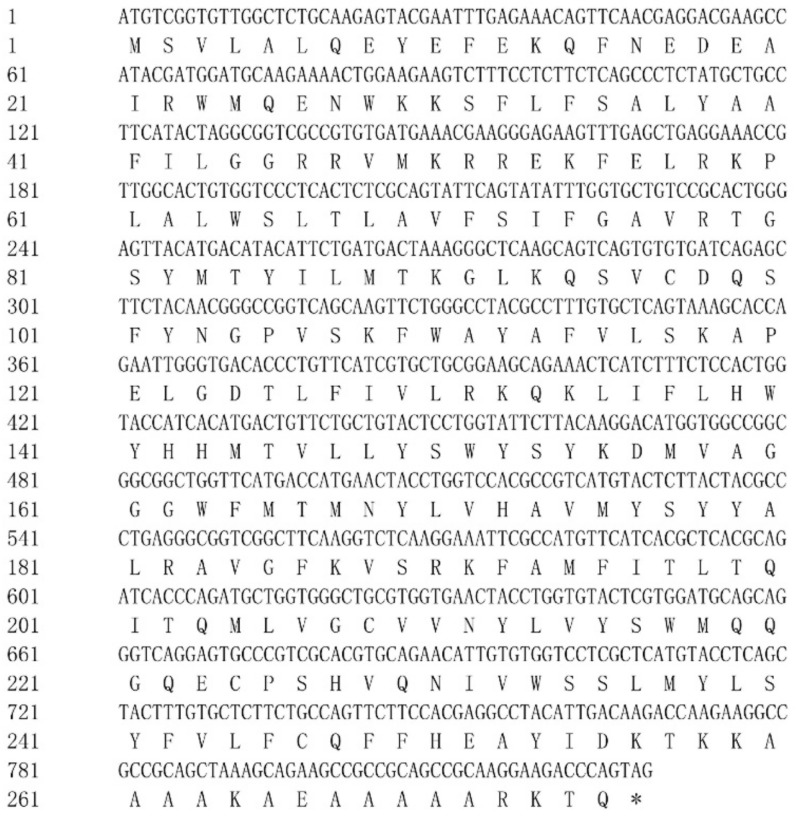
Nucleotide and deduced amino acid sequences of the elongation of very long-chain fatty acids protein 6 (Elovl6) in rainbow trout. * Represents the stop codon.

The rainbow trout *elovl6* cDNA contained an ORF of 828 bp encoding a putative protein of 275 amino acids (AA) (Figure 1). BLAST analysis of an amino acid sequence indicated that Elovl6 in rainbow trout shared high sequence identity with *Oncorhynchus kisutch* (99%), *Salmo salar* (97%), *Larimichthys crocea* (93%), *Labrus bergylta* (91%), *Cyprinus carpio* (91%), and *Danio rerio* (90%).

**Figure 2:**
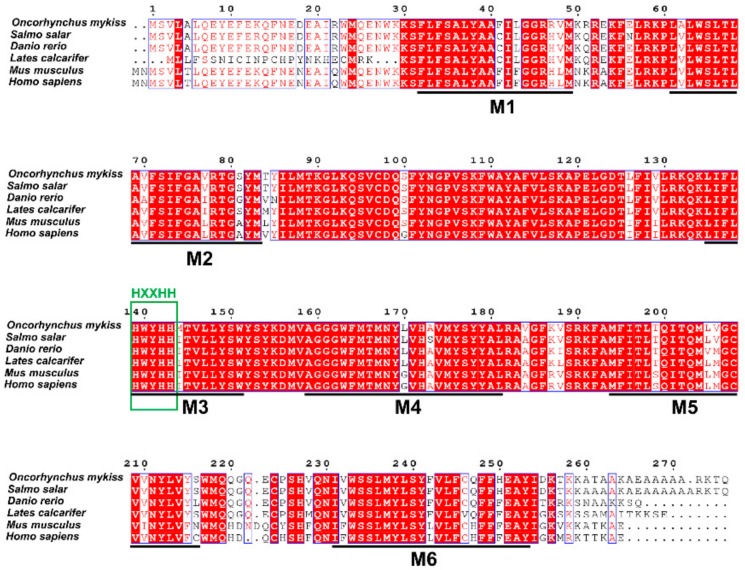
Amino acid alignment of Elovl6 amino acid sequence in rainbow trout with other species. The conserved HXXHH histidine box motif (green box) and putative membrane-spanning domains (M1–M6) are indicated. The blue boxes represent the conserved sequence. The identity shading is based on 87% identity threshold.

**Figure 3:**
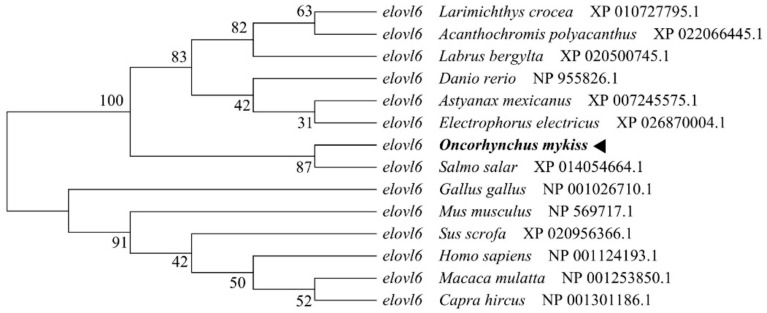
Phylogenetic tree of *elvol6* in rainbow trout and other vertebrate counterparts. The neighbor joining method in MEGA6 was used to construct the tree. The numbers represent the frequencies (%) with which the tree topology presented was replicated after 1000 iterations.

Rainbow trout Elovl6 protein possessed the characteristic features of Elovl family members, including histidine box (HXXHH) and six membrane-spanning domains (Figure 2). The deduced amino acid sequences showed an identity of 87% with the six species. Phylogenetic analysis clustered rainbow trout Elovl6 with other teleosts and mammals (Figure 3).

### 3.2. Tissue Distribution of Rainbow Trout Elovl6

**Figure 4:**
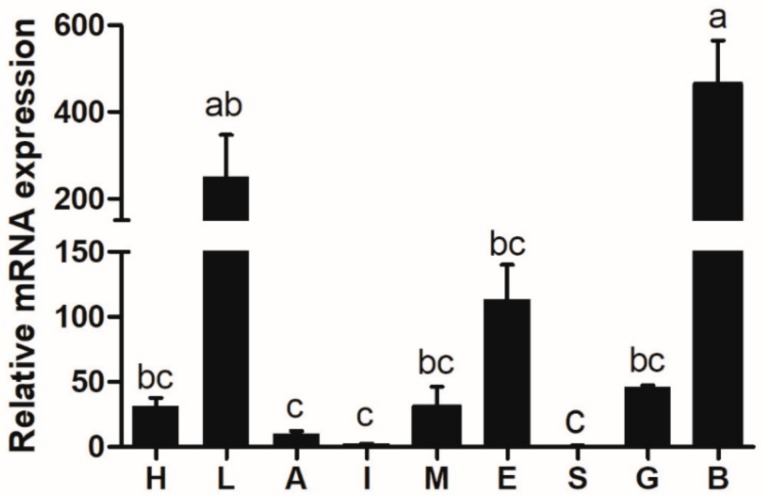
The transcriptional expression of *elovl6* in different tissues of rainbow trout. Heart (H), liver (L), adipose tissue (A), intestine (I), muscle (M), eye (E), spleen (S), gill (G), brain (B). The expression of *elvol6* in the spleen was selected as normalization. Data were presented as means ± S.E.M. (n = 3). Means that share the same superscript letter are not significantly different (*p* > 0.05).

The transcriptional expression of *elovl6* was determined in different tissues of rainbow trout, such as the brain, spleen, intestine, gill, liver, muscle, eye, heart, and adipose tissues (Figure 4). The high expression of *elovl6* in rainbow trout was observed in the brain, liver, and eye. Furthermore, the highest and lowest expression was detected in the brain and spleen, respectively.

### 3.3. Transcriptional Expression of Elovl6 in Response to Fatty Acids

#### 3.3.1. Expression of *Elovl6* in Response to Dietary Fatty Acids

**Figure 5:**
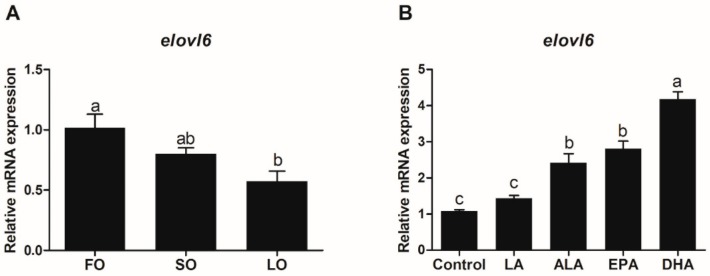
Effects of fatty acids on the transcriptional expression of *elovl6* in rainbow trout. (**A**) The *elovl6* expression in the liver of rainbow trout that were fed diets containing fish oil (FO), soybean oil (SO), and linseed oil (LO). (**B**) The expression of *elvol6* in hepatocytes of rainbow trout treated with 100 μM linoleic acid (LA), α-linolenic acid (ALA), eicosapentaenoic acid (EPA), and docosahexaenoic acid (DHA) for 12 h. FO and the control group were selected as normalization. Data were presented as means ± S.E.M. (n = 3). Means that share the same superscript letter are not significantly different (*p* > 0.05).

The transcriptional expression of *elovl6* in the liver of rainbow trout that were fed diets containing fish oil (FO), soybean oil (SO), or linseed oil (LO) was determined in the present study (Figure 5A). The expression of the *elovl6* gene in rainbow trout that were fed diets containing LO was significantly lower than that in rainbow trout that were fed diets containing FO (*p* < 0.05), and the expression in the SO group was lower than that in the FO group, but not significantly different (*p* > 0.05).

#### 3.3.2. Expression of *Elovl6* in Hepatocytes in Response to Fatty Acids

Incubation of hepatocytes from rainbow trout with fatty acids caused differences in *elovl6* expression (Figure 5B). Compared with the control group, the transcriptional expression of *elovl6* in hepatocytes treated with 100 μM α-linolenic acid (ALA), eicosapentaenoic acid (EPA), or docosahexaenoic acid (DHA) for 12 h was significantly increased (*p* < 0.05). The expression of *elovl6* in hepatocytes treated with 100 μM linoleic acid (LA) for 12 h was the lowest, while the highest expression of *elovl6* in hepatocytes was observed in the DHA group.

### 3.4. Transcriptional Expression of Elovl6 in Hepatocytes in Response to Insulin

**Figure 6:**
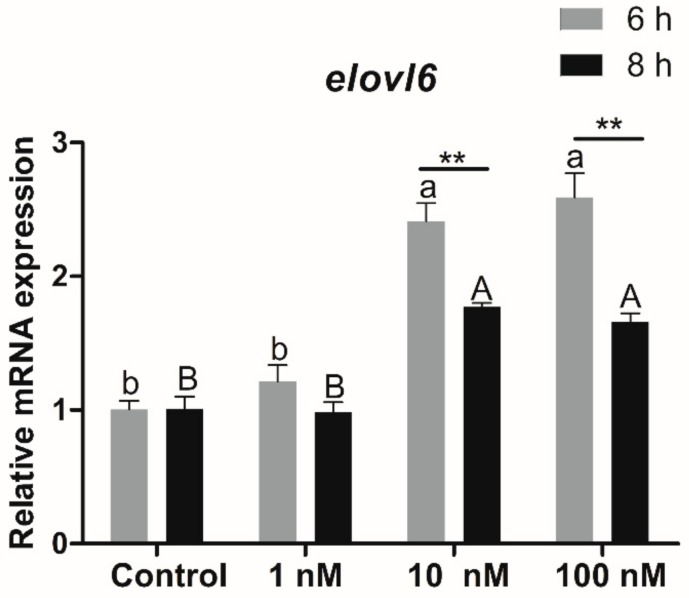
The transcriptional expression of *elovl6* in hepatocytes of rainbow trout treated with graded amounts of insulin. Control group was selected as normalization. Data were presented as means ± S.E.M. (n = 3). Capital letters show the differences in the 8 h group. Lowercase letters show the differences in the 6 h group. Means that share the same superscript letter are not significantly different (*p* > 0.05). ** *p* < 0.01.

To study the relationship between Elovl6 and insulin, the transcriptional expression of *elovl6* in hepatocytes in response to insulin was determined by qPCR. Compared with the control group, the expression of *elovl6* in hepatocytes of rainbow trout treated with 10 and 100 nM insulin for 6 or 8 h was significantly increased (*p* < 0.05), and the expression of *elovl6* in hepatocytes treated with 10 and 100 nM insulin for 6 h was significantly higher than that in the 8 h group (*p* < 0.01) (Figure 6).

### 3.5. Transcriptional Regulation of the Elovl6 in Response to Fatty Acids or Insulin

#### 3.5.1. Regulation of the *Elovl6* by Transcription Factors

**Figure 7:**
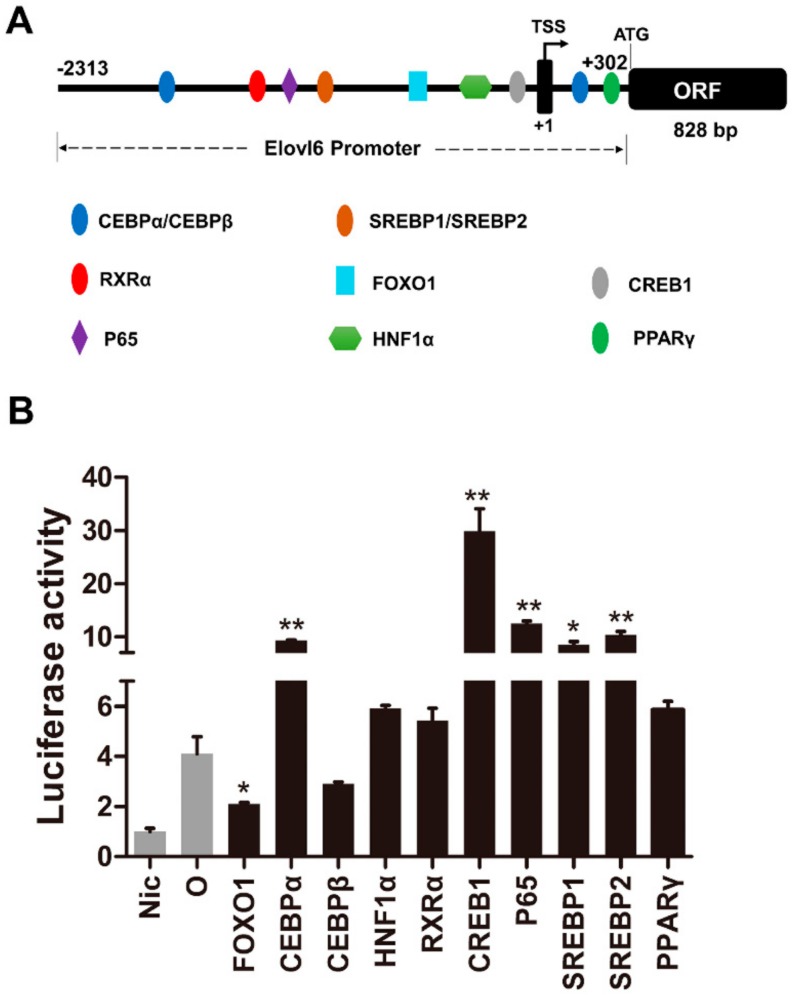
The transcription factors sites (**A**) and effects on the *elovl6* promoter (**B**) of rainbow trout in HEK 293T cells. The negative control Nic (pGL3-basic) was an empty plasmid with no promoter sequence. TSS: transcriptional start site. The promoter was co-transfected with plasmids of transcription factors FOXO1; CEBPα; CEBPβ; HNF1α; RXRα; CREB1; P65; SREBP1; SREBP2; and PPARγ, compared with the control group (O) which transfected with pGL3-*elovl6* promoter only, respectively. The luciferase activity in the Nic group was selected as normalization. Data were presented as means ± S.E.M. (n = 3). * *p* < 0.05; ** *p* < 0.01.

The upstream sequence of 2615 bp adjacent to the start of *elovl6* ORF was cloned as the promoter of *elovl6* in rainbow trout. The binding sites of transcription factors including CEBPβ, CEBPα, HNF1α, PPARγ, RXRα, SREBP1, SREBP2, CREB1, P65, and FOXO1 were predicted within the *elovl6* promoter region of rainbow trout by bioinformatics software (Figure 7A and Table 2). The dual-luciferase reporter assays in HEK 293T cells showed that the transcription factor CREB1 showed the largest activation of *elovl6* promoter activity (7.28-fold), and CEBPα, P65, SREBP1, and SREBP2 up-regulated the promoter activity of *elovl6* by 2.28-fold, 3.05-fold, 2.08-fold, and 2.52-fold, respectively (Figure 7B). Furthermore, the transcription factor FOXO1 significantly down-regulated the promoter activity of *elovl6*. However, the transcription factors CEBPβ, HNF1α, RXRα, and PPARγ showed no significant regulatory effect on the *elovl6* promoter of rainbow trout (Figure 7B).

#### 3.5.2. Transcriptional Expression of *Creb1* and *Foxo1* in Response to Dietary Fatty Acids

**Figure 8:**
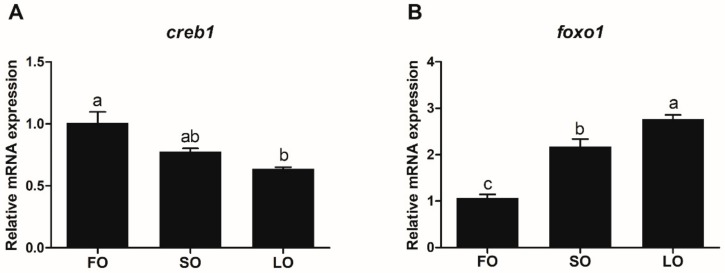
The transcriptional expression of *creb1* (**A**) and *foxo1* (**B**) in the liver of rainbow trout that were fed diets containing fish oil (FO), soybean oil (SO), and linseed oil (LO). The expression in FO was selected as normalization. Data were presented as means ± S.E.M. (n = 3). Means that share the same superscript letter are not significantly different (*p* > 0.05).

The transcriptional expression of *creb1* in the liver of rainbow trout that were fed diets containing SO and LO was lower than that in rainbow trout that were fed diets containing FO, and a significant difference was observed between the FO and LO groups (*p* < 0.05) (Figure 8A). The expression of *creb1* in the SO group was higher than that in the LO group, but there was no significant difference between the two groups (*p* > 0.05). The expression of the *foxo1* gene in the liver of rainbow trout that were fed diets containing with SO and LO was significantly higher than that in rainbow trout that were fed diets containing FO (*p* < 0.05), and the expression of *foxo1* in the LO group was significantly higher than that in the SO group (*p* < 0.05) (Figure 8B).

#### 3.5.3. Transcriptional Expression of *Creb1* and *Foxo1* in Hepatocytes in Response to Insulin

**Figure 9:**
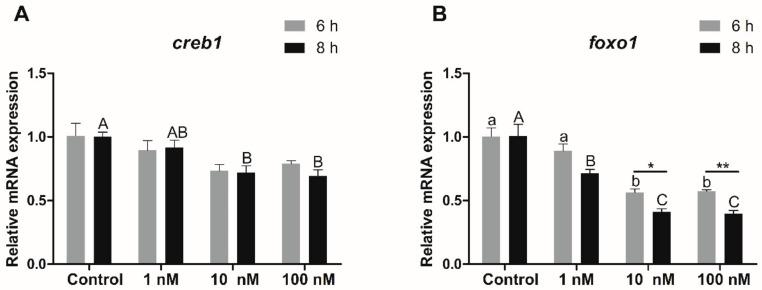
The transcriptional expression of *creb1* (**A**) and *foxo1* (**B**) in hepatocytes of rainbow trout treated with graded amounts of insulin. Control group was selected as normalization. Data were presented as means ± S.E.M. (n = 3). Capital letters show the differences in the 8 h group. Lowercase letters show the differences in the 6 h group. Means that share the same superscript letter are not significantly different (*p* > 0.05). * *p* < 0.05; ** *p* < 0.01.

The transcriptional expression of *creb1* in hepatocytes treated with insulin for 6 h showed no significant difference among the four groups (*p* > 0.05). Compared with the control group, the expression of *creb1* in hepatocytes of rainbow trout treated with 10 and 100 nM insulin for 8 h was significantly decreased (*p* < 0.05) (Figure 9A). There were no significant differences between the 6 h and 8 h groups (Figure 9A).

Compared with the control group, the transcriptional expression of *foxo1* in hepatocytes of rainbow trout treated with 10 and 100 nM insulin for 6 h was significantly decreased (*p* < 0.05). Furthermore, the transcriptional expression of *foxo1* in hepatocytes treated with 1, 10, and 100 nM insulin for 8 h was significantly decreased compared to the control group, and the transcriptional expression of *foxo1* in hepatocytes treated with 10 and 100 nM insulin for 6 h was significantly higher than that in the 8 h group (*p* < 0.05) (Figure 9B).

## 4. Discussion

As a microsomal enzyme, Elovl6 is specially positioned in the endoplasmic reticulum, which played a crucial role in the elongation of palmitate (PA) or palmitoleate (POA) to stearate or cis-vaccenic acid [13,14]. Previous studies in mammals demonstrated that Elovl6 is also involved in inflammatory and metabolic diseases, such as high-fat-diet-induced inflammation and insulin resistance [3,6,15]. However, few studies about Elovl6 in fish have been reported. In the present study, ORF of Elovl6 was cloned from the rainbow trout, which possessed the histidine box (HXXHH) and six membrane-spanning domains of Elovl proteins [16], and showed high identity (87%) with mammals and teleost. In rainbow trout, the high expression level of *elovl6* was observed in brain and liver tissues, which is consistent with the results in mice [2,4,5] and large yellow croaker [7].

In the present study, the expression of *elovl6* in the liver of rainbow trout that were fed diets containing FO was higher than that in the SO and LO groups. Our studies showed that fish oil in diets is richer in PA, POA, EPA, and DHA than soybean oil and linseed oil [7]. PA and POA are the substrates for the synthesis of C18:0 and C18:1n-7 [17]. Hence, the large amount of PA and POA in FO may promote the expression level of *elovl6* in rainbow trout to drive the synthesis of fatty acids [18]. A similar effect of PA on *elovl6* expression was also observed in large yellow croaker [7]. Furthermore, the transcriptional expression of *elovl6* in hepatocytes of rainbow trout treated with EPA and DHA was also higher than that in the ALA and LA groups, which may be another evidence for the high expression level of *elovl6* in the FO group. However, studies of large yellow croaker showed that *elovl6* expression in SO and LO groups was significantly higher than that in the FO group [7]. The results indicated that the *elovl6* of rainbow trout in response to dietary fatty acids is different from large yellow croaker. Rainbow trout, as a freshwater fish, generally has a relatively higher capacity for LC-PUFA synthesis from C18 PUFA (ALA or LA) in comparison to large yellow croaker (marine fish) where such a capacity is normally considerably lower. Hence, ALA and LA were considered as the essential fatty acids for rainbow trout, while the essential fatty acids for large yellow croaker were LC-PUFAs. Thus, the different responses to dietary fatty acids may be due to the difference in the requirements of essential fatty acids and evolutionary position between rainbow trout and large yellow croaker.

Previous studies in mammals have verified the crucial role of *elovl6* in insulin resistance [3,19]. Mice with a global deficiency for *elovl6* could protect against diet-induced insulin resistance [3], and *elovl6* specific deletion in the liver of mice ameliorated insulin resistance by reducing hepatic ceramide and protein phosphatase 2A activity [20]. However, studies on the relationship between *elovl6* and insulin in fish have not been reported to date. In the present study, the expression of *elovl6* in hepatocytes treated with 10 and 100 nM insulin for 6 or 8 h was significantly increased in rainbow trout. The results demonstrated that the transcriptional expression of *elovl6* in rainbow trout could be regulated by insulin. Previous studies have shown that insulin could promote the synthesis of lipids by the regulation of transcription factors related to lipid metabolism [21,22,23]. Thus, it could be speculated that the expression of some transcription factors would be changed when the hepatocytes are treated with the insulin, which may influence the expression of *elovl6* in rainbow trout. However, the precise mechanism is still not understood.

To explore the regulatory mechanism of *elovl6* expression in response to fatty acids or insulin, dual-luciferase reporter assay has been conducted with the *elovl6* promoter and transcription factors plasmids. Previous studies in mammals have reported that *elovl6* is consistently regulated by SREBP-1c,2 and PPARγ [2,5,18,24]. However, the transcription factor PPARγ showed no regulatory activity on the *elovl6* promoter in rainbow trout. In addition, CEBPα, P65, SREBP1, SREBP2, and CREB1 could up-regulate the promoter activity of *elovl6*, and CREB1 showed the largest activation of *elovl6* promoter activity in rainbow trout. CREB1 is a key intracellular transcription factor, which played a crucial role in regulating genes of lipid metabolism, such as PPARγ, ATGL, and UCP1 [25,26,27,28]. The similar activation of CREB1 and SREBP1 on *elovl6* has also been observed in large yellow croaker [7]. Furthermore, transcription factors CEBPβ, HNF1α, and RXRα showed significant activation of the *elovl6* promoter in large yellow croaker [7], but no regulatory effects were observed in rainbow trout. The difference in transcriptional regulation of *elovl6* by the transcription factors in two fishes may be due to the different requirements of essential fatty acids and evolutionary position. Surprisingly, the FOXO1, which links to insulin signaling and lipid metabolism [29,30,31,32], significantly down-regulated the activity of the *elovl6* promoter in rainbow trout. Previous studies highlighted the role of FOXO in integrating insulin signaling to hepatic glucose and lipid metabolism [33]. The present study showed the potential regulatory effect of FOXO1 on *elovl6* in fish, which may contribute to building up the interaction between the *elovl6* and insulin by FOXO1.

Studies on large yellow croaker have demonstrated that the transcription factors would influence the *elovl6* expression in response to fatty acids [7], and similar results have also been observed in mammals [18,24]. In the present study, the transcription factor CREB1 showed the most significant activation of *elovl6* promoter activity, and FOXO1 greatly down-regulated the promoter activity of *elovl6*. Thus, CREB1 and FOXO1 were chosen as the main transcription factor to further explain the regulation of *elovl6* expression in rainbow trout in response to fatty acids or insulin. The results showed that the expression of *creb1* in the liver of rainbow trout that were fed diets containing LO was significantly lower than that in the FO group and the low expression of *creb1* may make a decrease of *elovl6* expression in the LO group. Furthermore, the expression of *foxo1* in the SO and LO groups was significantly higher than that in the FO group, and *foxo1* could suppress the expression of *elovl6*. Thus, the decreased expression of *creb1* and increased expression of *foxo1* may explain the lower expression of *elovl6* in the SO and LO groups than in the FO group. Meanwhile, the *creb1* and *foxo1* expression in hepatocytes treated with insulin was also analyzed. The significant decrease in the expression of *creb1* was only observed in the 8 h group, but not dramatically. However, the dramatic decrease in expression of *foxo1* was observed both in 6 h and 8 h group. There is a double down-regulatory effect of *elovl6* by *creb1* and *foxo1* in the 8 h group, which may explain why the *elovl6* expression in hepatocytes treated with 10 and 100 nM insulin for 6 h was higher than that in the 8 h group. Thus, the dramatic decrease in expression of *foxo1* may mainly explain the increase in *elovl6* expression of hepatocytes in response to insulin. Furthermore, previous studies have shown that transcription factors including SREBP1, SREBP2, and CEBPα are also related to fatty acid metabolism and insulin signaling [12,34,35,36,37,38,39,40,41]. We speculated that the expression of *srebp1*, *srebp2*, and *cebpα* may also have effects on the transcriptional expression of *elovl6* in response to fatty acids or insulin. However, the speculation should be verified in the future. Overall, the transcription factors CREB1 and FOXO1 might be the potential targets to regulate the transcriptional expression of *elovl6* in rainbow trout in response to fatty acids or insulin. These results could help us to further understand the regulatory mechanism of nutrition and hormone in rainbow trout, and aid in the healthy and sustainable wide aquaculture of rainbow trout.

## 5. Conclusions

In conclusion, the cDNA of *elovl6* from rainbow trout was cloned in the present study, which showed the typical features of Elovl proteins. The transcriptional expression of *elovl6* in rainbow trout that were fed diets containing FO was higher than that in rainbow trout that were fed diets containing SO and LO, and the expression of *elovl6* was increased with the treatment of insulin. The transcription factor CREB1 could greatly up-regulate the activity of the *elovl6* promoter, and FOXO1 greatly down-regulated the activity. The differences in expression of *creb1* and *foxo1* may contribute to the increase or decrease of *elovl6* expression in rainbow trout in response to fatty acids or insulin.

## Figures and Tables

**Table 1:** Sequences of the primers for gene cloning, qPCR analysis, and promoter cloning.

Primer	Sequences 5′-3′	Primer Information
ORF-F	ATGTCGGTGTTGGCTCTGCAAG	Elovl6-ORF
ORF-R	CTACTGGGTCTTCCTTGCGGC	Elovl6-ORF
qElovl6-F	TCAACGAGGACGAAGCCATACGA	Elovl6 q-PCR
qElovl6-R	CCCAGTGCGGACAGCACCAAATA	Elovl6 q-PCR
qCREB1-F	AGGAGTCAGTGGACAGTGTGA	CREB1 q-PCR
qCREB1-R	TGCTGGTCTGGTAGATAGGGC	CREB1 q-PCR
qFOXO1-F	AACTCCCACAGCCACAGCAA	FOXO1 q-PCR
qFOXO1-R	CGATGTCCTGTTCCAGGAAGG	FOXO1 q-PCR
β-actin-F	ATCAGGGAGTGATGGTTGGGATG	β-actin q-PCR
β-actin-R	CTCGTAGATGGGTACTGTGTGGG	β-actin q-PCR
E6-P-F	GTAAACTGTTGCTGGAGATTCCGGAC	Elovl6 promoter
E6-P-R	GTTCACTGTGCGCTTTCCTGTAAACG	Elovl6 promoter
PCS2^+^-SREBP1-F	ATGAACTTGTCTTTTGACGATCAG	Expression plasmid
PCS2^+^-SREBP1-R	CTAGGCAGAGGTGACAGTGGTGC	Expression plasmid
PCS2^+^-SREBP2-F	ATGGACAGTAACGTTAGTGGGGAG	Expression plasmid
PCS2^+^-SREBP2-R	TCAGGAGGCCGCGATGGTG	Expression plasmid
PCS2^+^-CEBPα-F	ATGGAGCAACCAAACCTCTACGAG	Expression plasmid
PCS2^+^-CEBPα-R	TCACTGGCAGTTGGCCAATG	Expression plasmid
PCS2^+^-CEBPβ-F	TGGAAGTGGCCGGTTTCTACG	Expression plasmid
PCS2^+^-CEBPβ-R	CTAACCGGTGGCAGAAAGCAAG	Expression plasmid
PCS2^+^-HNF1α-F	ATGGAGGGAGAGGAGAGGAAAGG	Expression plasmid
PCS2^+^-HNF1α-R	CTACTGTGCGGTAGAGACCATCTGT	Expression plasmid
PCS2^+^-PPARγ-F	ATGCATATGATGTGTAGCAATTTTA	Expression plasmid
PCS2^+^-PPARγ-R	CTAGTAGAGGTCTCTCATGATCTCCT	Expression plasmid
PCS2^+^-RXRα-F	ATGACGCTGGAAATTCTGACATATT	Expression plasmid
PCS2^+^-RXRα-R	TTATGTCATTTGGTGGGGCG	Expression plasmid
PCS2^+^-CREB1-F	ATGACCATGGAGTCGGGAGC	Expression plasmid
PCS2^+^-CREB1-R	CTACTCAGATTTATGGCAGTACAGGTC	Expression plasmid
PCS2^+^-P65-F	ATGGATGGAATGTATGGATGGGG	Expression plasmid
PCS2^+^-P65-R	TAAGTCTGATGTCCGGACACGAA	Expression plasmid
PCS2^+^-FOXO1-F	ATGGCAGAATTACCACCGCCG	Expression plasmid
PCS2^+^-FOXO1-R	CTAGCCAGACACCCAGCTGTGTGTG	Expression plasmid

**Table 2:** The transcription factor binding sites predicted by software online.

Transcription Factor	Software	Position	Predicted Site
CEBPα/CEBPβ	JASPAR	−1417	TATTGCACCATA
		+52	AATTGCAAAATA
RXRα	JASPAR	−1155	TAACATTAAATAACTTTGG
P65	TF binding	−1081	GGAAACTCTC
SREBP1/SREBP2	JASPAR	−935	ATGGGGAGAT
FOXO1	JASPAR	−531	TGTAAACAAGA
HNF1α	JASPAR	−246	GGGTAATTGTTTAC
CREB1	JASPAR	−35	GGACGTCA
PPARγ	JASPAR	+104	TTGGTGGAGAGGGCC
